# Variation of Blood Metabolites of Brown Swiss, Holstein-Friesian, and Simmental Cows

**DOI:** 10.3390/ani10020271

**Published:** 2020-02-10

**Authors:** Anna Benedet, Marco Franzoi, Carmen L. Manuelian, Mauro Penasa, Massimo De Marchi

**Affiliations:** 1 Department of Agronomy, Food, Natural resources, Animals and Environment, University of Padova, Viale dell’Università 16, 35020 Legnaro (PD), Italy; mauro.penasa@unipd.it (M.P.); massimo.demarchi@unipd.it (M.D.M.); 2 Associazione Regionale Allevatori del Veneto (ARAV), Corso Australia 67/a, 35136 Padova, Italy; marco.franzoi89@gmail.com

**Keywords:** dairy cattle, milk mid-infrared spectroscopy, blood, metabolic profile, early lactation

## Abstract

**Simple Summary:**

Population-level phenotyping of blood metabolites is hardly achievable due to the limitation of reference analyses. Mid-infrared spectroscopy has recently been used to develop prediction models for major blood metabolites, allowing their determination on a large scale. The current study investigated the variation of blood β-hydroxybutyrate, non-esterified fatty acids, and urea nitrogen predicted from a large milk mid-infrared spectra database of Brown Swiss, Holstein-Friesian, and Simmental cows. Holstein-Friesian cows had the greatest concentrations of β-hydroxybutyrate and non-esterified fatty acids, and the lowest urea nitrogen in blood, which may underline an altered energy and nutritional status.

**Abstract:**

Serum metabolic profile is a common method to monitor health and nutritional status of dairy cows, but blood sampling and analysis are invasive, time-consuming, and expensive. Milk mid-infrared spectra have recently been used to develop prediction models for blood metabolites. The current study aimed to investigate factors affecting blood β-hydroxybutyrate (BHB), non-esterified fatty acids (NEFA), and urea nitrogen (BUN) predicted from a large milk mid-infrared spectra database. Data consisted of the first test-day record of early-lactation cows in multi-breed herds. Holstein-Friesian cows had the greatest concentration of blood BHB and NEFA, followed by Simmental and Brown Swiss. The greatest and the lowest concentrations of BUN were detected for Brown Swiss and Holstein-Friesian, respectively. The greatest BHB concentration was observed in the first two weeks of lactation for Brown Swiss and Holstein-Friesian. Across the first month of lactation, NEFA decreased and BUN increased for all considered breeds. The greatest concentrations of blood BHB and NEFA were recorded in spring and early summer, whereas BUN peaked in December. Environmental effects identified in the present study can be included as adjusting factors in within-breed estimation of genetic parameters for major blood metabolites.

## 1. Introduction

During early lactation, dairy cows experience severe metabolic changes due to the transition from gestation to milk production. These changes may cause the onset of negative energy balance (NEB), which makes cows more prone to metabolic disorders and health issues [1,2]. The NEB induces the mobilization of body reserves and the oxidation of non-esterified fatty acids (NEFA) in liver to produce energy. As a result, NEFA concentration in blood increases. If the maximum oxidizing capacity of liver is reached, ketone bodies such as β-hydroxybutyrate (BHB) are produced and released into the blood [3]. Thus, elevated serum concentrations of NEFA and BHB are key indicators of the mobilization of body energy reserves and the presence of hyperketonemia, defined as an abnormal concentration of circulating ketone bodies in cow blood, milk, and urine [2]. On the other hand, blood urea nitrogen (BUN) is an indicator of protein status and provides information on the rumen degradable protein intakes, and nitrogen utilization efficiency and excretion [4,5,6]. The concentration of BUN normally increases during the first weeks of lactation [7], being associated with an increased feed intake [8].

The availability of information on metabolic indicators is crucial to monitor the metabolic status of the herd and to promptly intervene in the case of metabolic issues. A common method to monitor the metabolic health and nutritional status of dairy cows is metabolic profiling, which is based on the determination of blood metabolites such as NEFA, BHB, and BUN. Nevertheless, blood sampling is invasive, stressful, and time-consuming, and the laboratory analyses are too expensive to be performed routinely. The semi-quantitative cow-side tests available to measure metabolites [2,9] are also laborious and expensive if used as a whole-herd screening tool.

Recently, calibration models to predict blood NEFA, BHB, and BUN from milk mid-infrared (MIR) spectra have been developed [7,10,11]. Those models had coefficients of determination from 0.39 (NEFA) to 0.90 (BUN), which makes it difficult to compare the absolute values obtained in different studies. However, these accuracies can be considered adequate to evaluate the differences among breeds in a multi-breed herd approach. The MIR calibration models have the advantage of providing routine data useful to monitor cow metabolic status, and avoiding invasive sampling and expensive analyses. Moreover, the availability of large data allows the investigation of phenotypic and genetic variation of blood metabolic traits at the population level. Thus, MIR spectroscopy is of particular interest to collect information on cow metabolic status, considering that test-day milk recording procedures are widely used to analyze milk gross composition. Currently, very few large-scale studies have investigated sources of variation of blood BHB, NEFA, and BUN in early-lactation cows of different breeds [12,13]. Therefore, the aim of the present research was to identify factors affecting blood BHB, NEFA, and BUN in multi-breed herds of Brown Swiss (BS), Holstein-Friesian (HF), and Simmental (SI) cows using data predicted from routine test-day milk MIR spectra.

## 2. Materials and Methods

### 2.1. Data Collection

The initial data comprised the spectra of individual milk samples collected in multi-breed herds of BS, HF, and SI cows during official monthly test-day milk recording between 2011 and 2018. The case study area was Bolzano Province, which is characterized by small-scale farms with traditional livestock feeding (forage or hay and concentrates). Moreover, approximately 20% of the herds are transhumed to mountain pastures in the summer season [14,15].

At the test-day, individual milk samples (50 mL) were collected during the morning or evening milking. They were immediately added with preservative (Bronysolv; ANA.LI.TIK Austria, Vienna, Austria) and processed at the milk laboratory of the South Tyrolean Dairy Association (Sennereiverband Südtirol, Bolzano, Italy) according to the International Committee for Animal Recording [16] recommendations. For each sample, fat (%), protein (%), casein (%), lactose (%), and milk urea nitrogen (MUN; mg/dL) were determined. Spectral information, containing 1060 infrared transmittance wavelengths in the region between 900 and 5000 cm^−1^, were stored using MilkoScan FT6000 (Foss Electric A/S, Hillerød, Denmark). Values of somatic cell count (SCC; cells/mL) were assessed by Fossomatic (Foss Electric A/S, Hillerød, Denmark) and converted to somatic cell score (SCS) through the formula SCS = 3 + log_2_(SCC/100,000).

### 2.2. Prediction of Blood Metabolites

Mid-infrared prediction models for blood metabolites developed by Benedet et al. [10] were applied to the stored milk spectra of the present study to predict blood concentrations of BHB, NEFA, and BUN. Briefly, between December 2017 and June 2018, 295 individual bovine blood and milk samples were collected from early-lactation BS, HF, and SI cows in northeast Italy. Reference analyses on blood samples for the determination of BHB, NEFA, and BUN concentrations were conducted in the Clinical Biochemistry Laboratory of the Experimental Zooprophylactic Institute of Lombardy and Emilia Romagna (IZSLER, Brescia, Italy) through an ILab 650 chemistry analyzer (Instrumentation Laboratory SpA, Milano, Italy) using a colorimetric assay for NEFA, an enzymatic kinetic colorimetric assay for BHB, and a urease test for BUN. Reference BHB values were log_10_-transformed to achieve a normal distribution. Prediction models were developed using partial least squares regression analysis after backward interval partial least squares procedure as described in Benedet et al. [10]. Coefficients of determination of prediction models in cross-validation were 0.64 for blood BHB, 0.54 for BUN, and 0.53 for blood NEFA [10].

### 2.3. Data Editing

The initial dataset was edited to ensure that multi-breed herds were under test-day recording scheme for at least four years during the study period (2011 to 2018). Moreover, only herds with at least two breeds and two cows per breed were considered. The first test-day record between five and 35 days in milk (DIM) of each lactation was retrieved from cows of parity one to 13. For each trait, values that deviated more than three standard deviations from the respective mean were considered inconsistent and removed from the dataset. After data editing, 43,201 test-day records of 24,566 cows in 765 herds were available for statistical analysis. Herd size ranged from four to 159 cows. Parity and DIM averaged 2.78 ± 1.76 and 20.70 ± 8.55 days, respectively. Number of herds, cows, and records for each breed and breed–herd combination, and descriptive statistics of parity, DIM, and herd size for each breed are summarized in Table 1.

### 2.4. Statistical Analysis

Sources of variation of the studied traits were investigated using the MIXED procedure of SAS software ver. 9.4 (SAS Institute Inc., Cary, NC, USA), according to the following linear mixed model:

y*_ijklmnop_* = μ + B*_i_* + P*_j_* + D*_k_* + M*_l_* + Y*_m_* + H*_n_* + (B × P)*_ij_* + (B × D)*_ik_* + (B × M)*_il_* + (P × D)*_jk_* + cow*_o_* + ε*_ijklmnop_*, (1)

where y*_ijklmnop_* is the dependent variable (milk yield, fat, protein, casein, and lactose percentage, MUN, SCS, log_10_-transformed blood BHB, NEFA, or BUN); μ is the overall intercept of the model; B*_i_* is the fixed effect of the *i*th breed of the cow (*i* = Brown Swiss, Holstein-Friesian, and Simmental); P*_j_* is the fixed effect of the *j*th parity of the cow (*j* = first, second, third, and fourth and later parities); D_k_ is the fixed effect of the *k*th class of stage of lactation of the cow (*k* = 1 to 6, the first being a class from five to 10 days, followed by five classes of five days each); M*_l_* is the fixed effect of the *l*th month of sampling (*l* = January to December); Y*_m_* is the fixed effect of the *m*th year of sampling (*m* = 2011 to 2018); H*_n_* is the fixed effect of the *n*th herd (n = 1 to 765); (B × P)*_ij_* is the fixed interaction effect between breed and parity; (B × D)*_ik_* is the fixed interaction effect between breed and stage of lactation; (B × M)*_il_* is the fixed interaction effect between breed and month of sampling; (P × D)*_jk_* is the fixed interaction effect between parity and stage of lactation; cow*_o_* is the random effect of the *o*th cow (*o* = 1 to 24,566); and ε*_ijklmnop_* is the random residual. A multiple comparison of means for the fixed effects was performed using Bonferroni’s test (*p* < 0.05).

## 3. Results and Discussion

### 3.1. Descriptive Statistics

The descriptive statistics of the predicted blood metabolites and milk traits are summarized in Table 2. The average BHB value in the present study (0.65 mmol/L) was within the range reported for HF cows (0.54 mmol/L [7] to 0.80 mmol/L [17]). The mean value for NEFA (0.36 mmol/L) was lower than the one observed by Luke et al. [7] (0.49 mmol/L) in HF and similar to that obtained by Djoković et al. [18] (0.38 mmol/L) in early-lactation SI cows. The average BUN (2.87 mmol/L) was lower than previous findings in early-lactation cows [7,19]. The lower blood metabolite concentration in the present study compared with other studies may be due to differences in the management of the herds. In our study, multi-breed farms were located in a mountain area characterized by traditional feeding and lower productivity than herds in the plain. As a matter of fact, in the current analysis, the percentage of records with concentration of blood metabolites outside the normal range was very low. Indeed, the percentage of records suggesting hyperketonemia (BHB ≥ 1.2 mmol/L [2]) was 2.4%. Additionally, 5.3% of the data exhibited NEFA concentration ≥ 0.70 mmol/L, which is considered a critical threshold to identify cows with high body reserves mobilization [2]. Additionally, about 10% of samples showed abnormal concentrations of BUN (< 1.7 mmol/L or > 6.8 mmol/L [6,20]), suggesting possible unbalanced diets to fed cows. Overall, these findings indicate that herds of the present study had low prevalence of metabolic disorders.

Bearing in mind that only the first month of lactation was investigated here, average milk yield was slightly greater, fat percentage was similar, and SCS and protein and casein percentages were slightly lower than those reported in multi-breed herds of northeast Italy [21,22,23]. As indicated in previous investigations [21,22], SCS was the most variable milk trait, followed by MUN and milk yield (Table 2).

### 3.2. Breed Effect

Least squares means of predicted blood metabolites, milk yield, and composition traits for BS, HF, and SI breeds are shown in Table 3. Small differences (*p* < 0.05) were observed among the three breeds for BHB concentrations in blood; the greatest (0.65 mmol/L) and the lowest (0.62 mmol/L) concentrations were detected for HF and BS, respectively, whereas SI presented an intermediate value (0.63 mmol/L). Moreover, HF had the greatest NEFA (0.42 mmol/L) and the lowest BUN concentrations (2.67 mmol/L), which could suggest that HF cows are more prone to incur metabolic disorders than other breeds. In fact, elevated BHB and NEFA, and low BUN commonly indicate an insufficient energy and protein intake due to the diet or inability of the animal to cope with the NEB that characterizes the first month of lactation [1,6]. Brown Swiss and SI showed the same concentration of NEFA (0.35 mmol/L), but different BUN concentrations (3.18 vs. 2.80 mmol/L, respectively; *p* < 0.05). These results partially agreed with Urdl et al. [13], who reported similar BUN concentrations among BS, HF, and SI breeds, and the greatest BHB concentration in early-lactation BS and HF cows. Moreover, the same authors did not observe a significant breed effect on NEFA, concluding that breed is less important than energy intake or milk production on blood metabolites. In contrast, our findings suggested that breed is an important source of variation of early-lactation blood metabolites, even if the reasons of some differences among breeds are still unclear. For instance, the dual-purpose SI was expected to be the best to cope with the energy stress of milk production and, thus, we expected the lowest NEFA and BHB concentrations for this breed. However, SI is probably characterized by a different, but not well-known, metabolic pathway following the slower increase of milk production toward the peak compared with specialized dairy breeds (HF and BS).

Holstein-Friesian yielded more milk (32.83 kg/d; *p* < 0.05) than BS and SI, and BS had the greatest percentages of fat (4.13%), protein (3.33%), and casein (2.61%; *p* < 0.05). In general, SI cows were intermediate for these traits, except for fat percentage, which was very similar to fat percentage of HF cows. The greatest (20.80 mg/dL; *p* < 0.05) and lowest (16.42 mg/dL; *p* < 0.05) MUN contents were estimated for BS and HF, respectively, reflecting the same situation depicted for BUN concentration (*p* < 0.05; Table 3). The lowest SCS was observed for SI breed (1.74; *p* < 0.05) and the greatest for HF (2.24; *p* < 0.05). Overall, although considering the whole lactation, similar trends for milk production and composition traits of BS, HF, and SI cattle breeds were obtained in previous Italian studies [21,23,24]. In fact, Italian HF is the most productive breed, with the lowest milk composition, Italian BS has the most favorable milk composition, and Italian SI has the lowest SCS and intermediate values for milk traits.

### 3.3. Interaction Effects

**Figure 1:**
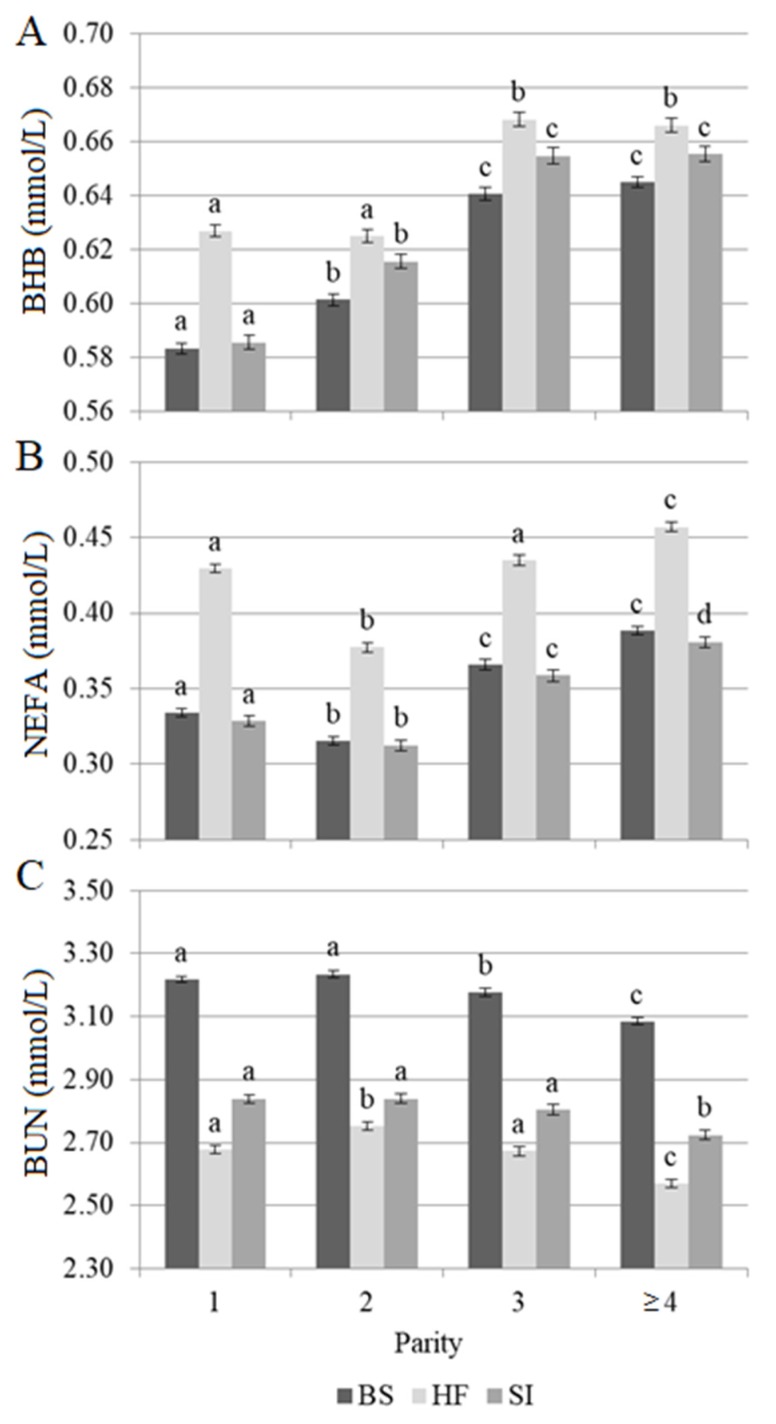
Least squares means of predicted (**A**) blood β-hydroxybutyrate (BHB), (**B**) blood non-esterified fatty acids (NEFA), and (**C**) blood urea nitrogen (BUN) across parity of Brown Swiss (BS), Holstein-Friesian (HF), and Simmental (SI) cows in multi-breed herds. Different superscript letters indicate significantly different least squares means within breed, according to Bonferroni’s test (*p* < 0.05). Log_10_-transformed BHB was the trait analyzed as dependent variable in the mixed model; however, for clarity of presentation, least squares means were back-transformed to present results in mmol/L. Vertical bars indicate the standard error.

Least squares means of predicted blood metabolites of BS, HF, and SI cows for parity effect are depicted in Figure 1. The concentrations of BHB and NEFA were generally greater in the third and later compared with the first and second lactation for all considered breeds (*p* < 0.05). Focusing on HF, primiparous cows had similar or greater BHB and NEFA than cows in second lactation. For BS and SI, the lowest BHB concentrations were observed for primiparous cows (*p* < 0.05). On the other hand, the trend of NEFA across parity in BS and SI was similar to that of HF, i.e., NEFA was greater in the first than second lactation (*p* < 0.05). This trend was previously reported by Mäntysaari et al. [25] for Nordic Red cows. The increase of blood BHB and NEFA concentrations with parity is generally expected [26,27]. However, elevated concentrations of BHB and NEFA in the blood of first-lactation cows could be due to their concurrent energy demands for growth and lactogenesis or a worse energy status than multiparous cows around calving [28,29]. Overall, BUN decreased slightly across parities in all considered breeds (Figure 1). In general, we expected no significant association or an opposite weak trend of BUN [29] or MUN across parity [30].

**Figure 2:**
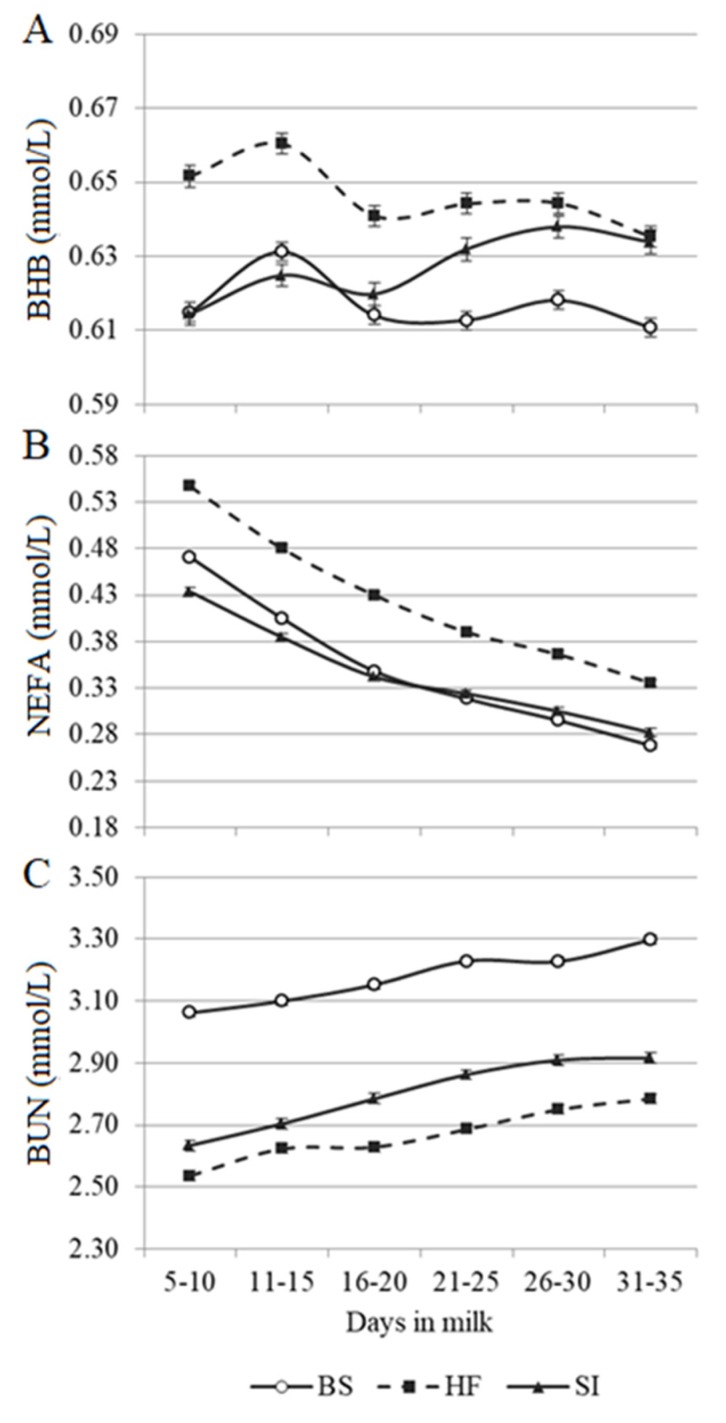
Least squares means of predicted (**A**) blood β-hydroxybutyrate (BHB), (**B**) blood non-esterified fatty acids (NEFA), and (**C**) blood urea nitrogen (BUN) in the first month of lactation of Brown Swiss (BS), Holstein-Friesian (HF), and Simmental (SI) cows in multi-breed herds. Log_10_-transformed BHB was the trait analyzed as dependent variable in the mixed model; however, for clarity of presentation, least squares means were back-transformed to present results in mmol/L. Vertical bars indicate the standard error.

Predicted blood metabolites across the first month of lactation for the three breeds are depicted in Figure 2. Although with greater concentrations, HF exhibited a comparable pattern for BHB than BS, with a peak between 11 and 15 DIM and a fluctuating decline thereafter. On the other hand, SI exhibited a nonlinear, but generally consistent, increase across early lactation. Considering NEFA and BUN, a decreasing and an increasing linear trend was observed, respectively (Figure 2). Holstein-Friesian had the greatest NEFA concentrations, whereas BS and SI had similar NEFA concentrations from 5 to 35 DIM. The greatest and the lowest BUN concentrations were observed for BS and HF, respectively, with intermediate values for SI. Overall, trends of predicted blood metabolites detected in the current study agreed with previous findings conducted on HF cows [8,31,32]. Although a stationary [33] or increasing [8] BHB trend was previously observed in the first month of lactation of HF cows, there is a lack of information for BS and SI breeds in the literature. The opposite direction of NEFA and BHB patterns between five and 15 DIM may be explained by the different use of these metabolites by liver and body tissues [29]. The increase of BUN concentration after calving is likely to be associated with the progressive increase of feed intake [8].

**Figure 3:**
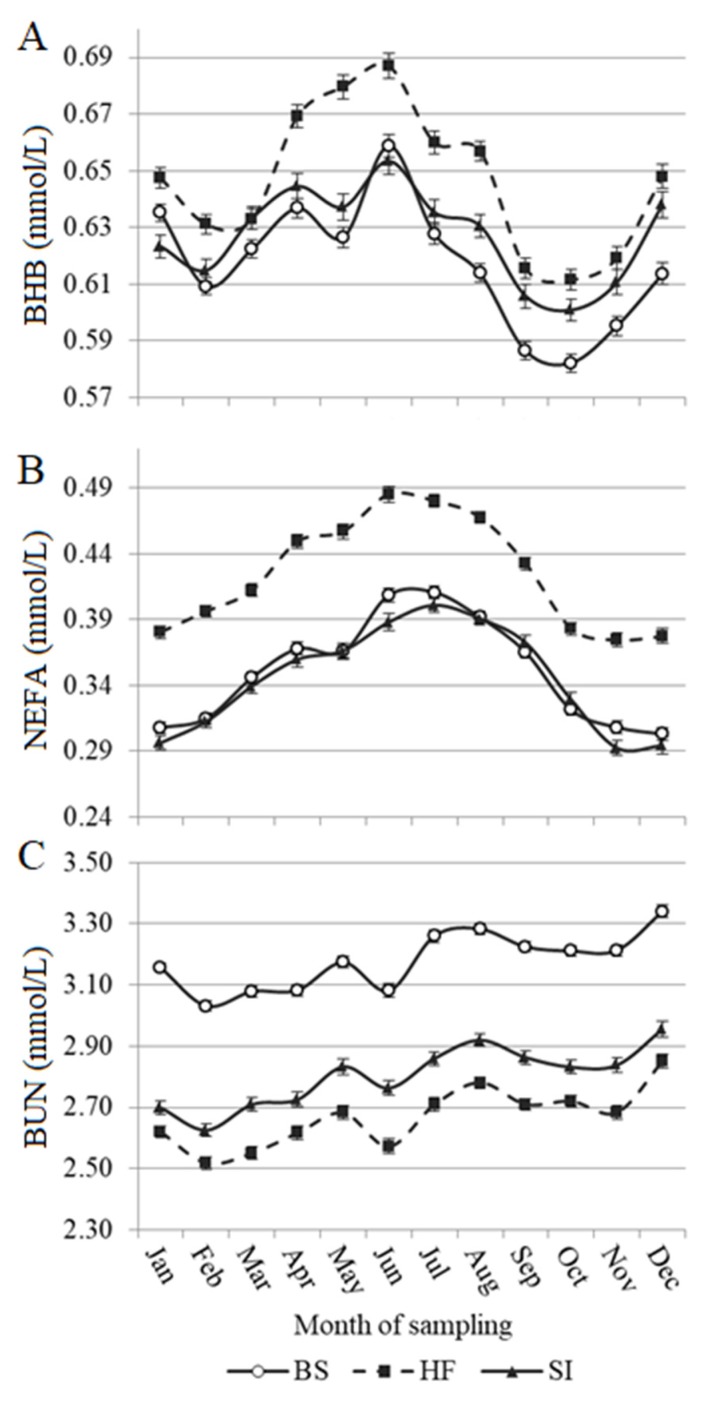
Least squares means of predicted (**A**) blood β-hydroxybutyrate (BHB), (**B**) blood non-esterified fatty acids (NEFA), and (**C**) blood urea nitrogen (BUN) across months of sampling in Brown Swiss (BS), Holstein-Friesian (HF), and Simmental (SI) cows in multi-breed herds. Log_10_-transformed BHB was the trait analyzed as dependent variable in the mixed model; however, for clarity of presentation, least squares means were back-transformed to present results in mmol/L. Vertical bars indicate the standard error.

Least squares means of blood metabolites across months of sampling for HF, BS, and SI breeds are depicted in Figure 3. The number of calvings per season was quite equilibrated: 28% in winter, 21% in spring, 26% in summer, and 25% in fall. Concentration of blood BHB fluctuated across months of sampling, peaking in early summer, and then decreasing toward fall. Similarly, but with a more linear trend, NEFA concentrations increased from January to June, and then decreased until December. The increased fat mobilization denoted by the increase of BHB and NEFA concentrations may have been caused by the lower feed intake caused by summer heat stress, and the beginning of grazing season with the correlated metabolic changes [34]. Moreover, the same reasons could have affected trends of BUN across months for all studied breeds (Figure 3). In fact, following a slightly increasing trend from February to May, especially for SI and HF cows, BUN dropped in June and then increased again during summer. The influence of pasture and temperature on dry matter intake could have caused a negative protein balance in early summer [30,34].

## 4. Conclusions

Significant differences were observed for blood metabolites in the most important Italian cattle breeds. The greatest concentrations of BHB and NEFA in blood were detected for HF cows, followed by SI and BS. Conversely, the greatest and the lowest BUN were observed in BS and HF, respectively. Blood BHB and NEFA concentrations increased with parity. The greatest BHB was observed in the first two weeks of lactation, except for SI, which exhibited a small increase across early lactation. In all breeds, NEFA declined and BUN increased in the first month of lactation. The maximum concentrations of blood BHB and NEFA were recorded during spring and early summer, whereas BUN generally increased from spring to late fall. Environmental effects identified in the present study can be considered as adjusting factors in within-breed estimation of genetic parameters for major blood metabolites.

## Figures and Tables

**Table 1:** Number of herds, cows, and records by breed and breed combination, and descriptive statistics of parity, DIM, and herd size by breed. Breeds are: Brown Swiss (BS), Holstein-Friesian (HF), and Simmental (SI).

Item	Herds	Cows	Records	Parity	DIM (Days)	Herd Size (Cows)
Breed	n	n	n	mean ± SD	mean ± SD	mean ± SD
BS	597	9992	17,600	2.86 ± 1.81	20.75 ± 8.53	16.4 ± 15.05
HF	549	8203	13,854	2.61 ± 1.62	20.71 ± 8.58	14.94 ± 16.65
SI	487	6371	11,747	2.86 ± 1.85	20.61 ± 8.55	13.08 ± 15.40
Breed combination						
BS + HF	278	9633	16,849	-	-	-
BS + SI	216	4823	8683	-	-	-
HF + SI	168	5943	10,585	-	-	-
BS + HF + SI	103	4167	7048	-	-	-

**Table 2:** Mean, standard deviation (SD), coefficient of variation (CV), minimum, and maximum of predicted blood metabolites, milk yield, and composition traits of early-lactation cows in multi-breed herds (43,201 observations).

Trait ^1^	Mean	SD	CV (%)	Minimum	Maximum
Blood metabolite (mmol/L)					
BHB ^2^	0.65	0.23	35.05	0.19	2.78
NEFA	0.36	0.19	53.27	0.01	1.32
BUN	2.87	0.84	29.37	1.20	11.53
Milk trait					
Milk yield (kg/d)	30.95	6.98	22.54	9.10	56.50
Fat (%)	4.10	0.74	17.93	1.68	6.87
Protein (%)	3.28	0.35	10.77	2.18	4.52
Casein (%)	2.57	0.27	10.54	1.65	3.51
Lactose (%)	4.81	0.17	3.51	4.06	5.36
MUN (mg/dL)	18.87	7.17	38.00	0.10	43.40
SCS	2.08	1.94	93.12	−3.64	9.61

^1^ BHB = β-hydroxybutyrate; NEFA = non-esterified fatty acids; BUN = blood urea nitrogen; MUN = milk urea nitrogen; SCS = somatic cell score. ^2^ For clarity of presentation, log_10_-transformed BHB values were back-transformed to present results in mmol/L.

**Table 3:** Least squares means ^1^ (standard error) of predicted blood metabolites, milk yield, and composition traits of Brown Swiss, Holstein-Friesian, and Simmental cows in multi-breed herds.

Trait ^2^	Brown Swiss	Holstein-Friesian	Simmental
Blood metabolite (mmol/L)			
BHB ^3^	0.62 (0.001) ^a^	0.65 (0.002) ^c^	0.63 (0.002) ^b^
NEFA	0.35 (0.002) ^a^	0.42 (0.002) ^b^	0.35 (0.002) ^a^
BUN	3.18 (0.01) ^c^	2.67 (0.01) ^a^	2.80 (0.01) ^b^
Milk trait			
Milk yield (kg/d)	29.28 (0.06) ^a^	32.83 (0.07) ^b^	29.40 (0.07) ^a^
Fat (%)	4.13 (0.007) ^b^	4.06 (0.008) ^a^	4.05 (0.009) ^a^
Protein (%)	3.33 (0.003) ^c^	3.16 (0.003) ^a^	3.30 (0.004) ^b^
Casein (%)	2.61 (0.002) ^c^	2.47 (0.003) ^a^	2.58 (0.003) ^b^
Lactose (%)	4.82 (0.002) ^b^	4.77 (0.002) ^a^	4.81 (0.002) ^b^
MUN (mg/dL)	20.80 (0.07) ^c^	16.42 (0.08) ^a^	18.47 (0.09) ^b^
SCS	2.08 (0.02) ^b^	2.24 (0.02) ^c^	1.74 (0.03) ^a^

^1^ Least squares means with different superscript letters within a row are significantly different according to Bonferroni’s test (*p* < 0.05). ^2^ BHB = β-hydroxybutyrate; NEFA = non-esterified fatty acids; BUN = blood urea nitrogen; MUN = milk urea nitrogen; SCS = somatic cell score. ^3^ Log_10_-transformed BHB was the trait analyzed as dependent variable in the mixed model; however, for clarity of presentation, least squares means were back-transformed to present results in mmol/L.
